# Influences of nitrogen inputs on nematode populations under highbush blueberry

**DOI:** 10.21307/jofnem-2020-056

**Published:** 2020-07-06

**Authors:** Thomas Forge, David Ehret, Aime Messiga, Martine Dorais

**Affiliations:** 1 Agriculture and Agri-Food Canada, Summerland Research and Development Centre, 4200 Hwy 97, Summerland, BC V0H 1Z0, Canada; 2 Agriculture and Agri-Food Canada, Agassiz Research and Development Centre, 6947 Hwy 7, PO Box 1000, Agassiz, BC V0M 1A0, Canada; 3 Centre de Recherche et d’Innovation sur les végétaux (CRIV), Dép. de Phytologie, Faculté des sciences de l'agriculture et de l'alimentation, Pavillon Envirotron, Université Laval, Québec G1V 0A6, Canada

**Keywords:** Blueberry, Cultural management, Fertilization, Nematode ecology, Population dynamics, Soil food web, *Vaccinium*

## Abstract

This study examined the effects of nitrogen fertilization on populations of *Rotylenchus robustus*, *Pratylenchus crenatus*, and *Paratrichodorus renifer*, and indices of free-living nematode community structure, in relation to highbush blueberry production in British Columbia, Canada. The field experiment was established in fall of 2008 with six replicate plots of each of four experimental N fertilization treatments: 0, 100, 150, and 200% of the annual application rate recommended for conventional blueberry production in the region. Nematode populations were quantified annually from 2009 through 2015, and then nematode populations and root biomass were quantified at seven sample dates from 2016 through 2019. Population densities of *R. robustus* were consistently greater in the 100% treatment than in the 0, 150, and 200% treatments which did not differ from each other. Population densities of *P. crenatus* were consistently greater in the 150% treatment than in the 0, 100%, and 200% treatments. The nematode structure index and two indices of diversity declined monotonically with N fertilizer rate, indicating broader changes in the soil food web that could have had indirect, feedback effects on population dynamics of the plant-parasitic nematodes.

Highbush blueberry (*Vaccinium corymbosum* L.) is an economically important crop in British Columbia (BC) and the Pacific Northwest of North America. In BC, over 12,000 ha of blueberry fields yielded an average of 76,750 tons of marketable fruit over the 2014 to 2015 period (Statistics Canada, 2019). Several groups of plant-parasitic nematodes have been found to be associated with highbush blueberry in the region, with *Pratylenchus* and *Paratrichodorus* nematodes being the most frequently found genera (Zasada et al., 2010). Subsequent research assessed the pathogenicity of *Paratrichodorus renifer*, the most common *Paratrichodorus* species in blueberry plantations in BC (Forge et al., 2012), and documented the host parasite-relationship of *Pratylenchus crenatus* with highbush blueberry and weeds commonly found in blueberry plantations in the region (Zasada et al., 2017).

Optimal soil conditions and production practices for highbush blueberry differ from other perennial fruit crops grown in the region: The optimal soil pH for blueberry production, 4.5 to 5.2, is lower than for most other crops. Most commercial blueberry production also involves the use of sawdust mulch that helps to maintain low soil pH, suppress weeds, and moderate soil temperature and moisture regimes (BCMAL, 2020). Due in-part to the use of such carbon-rich mulches, most conventional blueberry production in the region depends on supplemental application of nitrogen (N) fertilizers to balance N immobilization and maintain productivity (Ehret et al., 2014; Messiga et al., 2018). The effects of N fertilizer application rates on blueberry productivity, fruit quality, and soil N dynamics have been the focus of previous research in the region (Ehret et al., 2014; Messiga et al., 2018).

Nitrogen fertilization may also influence diseases and pest populations that could in-turn have longer-term negative feedback effects on productivity of blueberry plantations. However, little is known of the effects of N fertilization on pests of blueberry. Previous research on the influences of N fertilization on genera or species of plant-parasitic nematodes of agronomic significance was focused primarily on natural grassland, pasture, forage, or cereal production systems (Dmowska and Ilieva, 1995; Todd, 1996; Sarathchandra et al., 2001; Verschoor et al., 2001; Forge et al., 2005a), and most of such studies reported an increase in population densities with increasing N fertilization rates. With respect to horticultural crops, Forge et al. (2019) reported an increase in population densities of *Mesocriconema xenoplax* with N fertilization rates to grapevines. Collectively, these results are consistent with theory and observations on responses of other invertebrate herbivores to plant N enrichment (Mattson, 1980; Mahdavi-Arab et al., 2014). In contrast, Azpilicueta et al. (2014) reported that N fertilization decreased population densities of *Pratylenchus* sp. in apple orchard plots relative to non-fertilized plots.

Nitrogen fertilization has also been commonly observed to suppress certain populations of free-living soil nematodes, particularly omnivores and predators in the Dorylaimida with ‘persister’ or K-selected traits, resulting in reduced indices of diversity and food web structure such as the nematode community structure index (e.g. Ferris et al., 2001; Forge et al., 2005b; Wang et al., 2006; Azpilicueta et al., 2014; Pan et al., 2015; Song et al., 2016). Such changes in soil food web structure have been theoretically associated with reduced capacity of the soil food web to provide pest-suppressive services, facilitating increased population densities of plant-parasitic species (Sánchez-Moreno and Ferris, 2007; McSorley et al., 2008; Ferris et al., 2012).

In 2009, a field experiment was established in BC to assess the multi-year effects of a range of N fertilizer application rates and application methods on blueberry productivity, fruit quality, soil chemical properties, and potential for nitrate leaching (Ehret et al., 2014; Messiga et al., 2018). This experiment presented an opportunity to also assess the effects of N fertilization on nematode populations under blueberry. Specifically, the objective of this research was to determine the effects of N fertilization on population densities of three species of plant-parasitic nematodes (*Rotylenchus robustus, Pratylenchus crenatus*, and *Paratrichodorus renifer*), root biomass, and indices of free-living soil nematode diversity and food web structure, under highbush blueberry.

## Materials and methods

### Study site

The 0.15-ha field of northern highbush blueberry (*Vaccinium corymbosum* L.) cv ‘Duke’ was planted in October, 2008 at the Agriculture and Agri-Food Canada, Agassiz Research and Development Centre, in Agassiz, British Columbia (lat. 49^°^14´33˝N, long. 121^°^45´35˝W). Over the preceding decade, the site was used for mixed-cropping, alternating between vegetable and forage crops. Soil at the site is a moderately well-drained Monroe series (eluviated eutric Brunisol) silt loam (Luttmerding and Sprout, 1967). At planting, soil organic matter was 5.27%, with 3.24% total organic carbon and 0.26% total N. The field was plowed and disked in the spring prior to planting and amended with elemental sulphur (0-0-0-90S; TerraLink Horticulture Inc., Abbotsford, BC) at a rate of 1,120 kg ha^–1^ to lower soil pH initially from 5.6 to 5.0. The field was then subsoiled and a bed shaper was used to create six blocks of five 1-m wide × 0.2-m high × 13 m long raised beds, with 3 m spacing between the row centers of the five beds in each block. Each bed was planted with 14 blueberry plants, with a 0.91 m spacing between plants. The plants were 2-yr-old container stock from a commercial nursery (JRT Nurseries, Abbotsford, BC). Approximately 8 cm of fresh western hemlock (*Tsuga heterophylla* Sarg.) and Douglas-fir (*Pseudotsuga menziesii* Franco) sawdust was applied on top of the beds as mulch, immediately after planting and reapplied in spring 2010, 2012, 2014, 2016, and 2018. All areas between and around the beds were seeded with a mix of 30% fescue and 70% perennial ryegrass (Alleyway Agricultural Mix, Richardson Seed, Abbotsford, BC).

### Experimental design

Each bed was divided into two treatment plots of seven plants each, resulting in 10 plots in each of the six blocks. A total of 10 different fertilizer N treatments were applied to each of the six blocks of 10 plots as described in detail in Ehret et al. (2014) and Messiga et al. (2018). Only four of those treatments were sampled as part of this study. These treatments consisted of an unfertilized control (0N) and three levels of N applied at 50, 100, and 150% of rates recommended in the British Columbia Berry Production Guide (BCMAL, 2020) from 2009 through 2012. Commencing in 2013, the annual application rates in these three treatments shifted to 100, 150, and 200% of the rates recommended in the Production Guide due to realization that many growers in the region were using rates in excess of those recommended in the Production Guide (Table 1). By 2019, cumulative N inputs for these three treatments were 1,020, 1,573, and 2,126 kg N ha^–1^, and these four treatments are hereafter referred to as the 1N, 1.5N, and 2N treatments (Table 1). The N was applied as granular ammonium sulphate (21-0-0), broadcast on the surface of the sawdust mulch around the dripline of each plant in three equal split applications over an eight-week period each spring beginning at bud break (late April to early May).

**Table 1.** Actual nitrogen application rates by year for the three fertilizer treatments sampled in this study, in relation to rates recommended for blueberry production in British Columbia (BCMAL, 2020).

		Nitrogen application rates (kg N ha^–1^)					
	2009	2010	2011	2012	2013	2014	2015-2019
1N	11	14	25	39	100	111	144
1.5N	22	29	50	79	151	167	215
2N	32	43	75	118	201	222	287
Recommended	22	29	50	79	100	111	144

**Note:** Fertilizer was broadcast to the mulched area directly under blueberry plants as granular ammonium sulphate (21-0-0).

All plots were irrigated with two drip lines (DLT Heavywall Dripperline, Netafim, Fresno, CA) running down both sides of each bed, with each dripline located 20 cm out from center of the bed. The drip lines had 1 L h^–1^ emitters spaced every 0.45 m. All plots were irrigated throughout the season depending on soil moisture conditions. Soil moisture tension and soil water content were monitored with granular matrix sensors (Watermark Model 900M, Irrometer Co., Riverside, CA) and EC-5 sensors (Decagon Devices Inc. Pullman, WA), respectively, in one block. Irrigation was initiated as needed during the growing season to maintain volumetric water contents between 0.22 and 0.27 m^3^/m^3^. Based on foliar analyses, plants were also fertilized with, on average, 11.5 kg ha^–1^ of P_2_O_5_ (triple superphosphate; 0-45-0) and 15.8 kg ha^–1^of K_2_O (K-Mag; 0-0-21.5) applied as a broadcast band around each plant’s dripline in May and August of each year.

### Nematode sampling and analyses

Composite soil samples were taken from each plot in September of 2009 and yearly from 2011 through 2015; in both 2016 and 2017, samples were taken in April, June, and September, and in 2019 samples were taken in June. At each sampling date, two 2-cm diameter × 30-cm deep cores were taken from around the base of each of the five central plants in each plot of seven plants. The resulting 10 cores were combined to form a single composite sample representing the plot. Sawdust mulch was not included in the cores. The 10 cores were taken from an approximately 30 cm radius around the base of the five measurement bushes in each plot (two cores per bush) as follows: two cores were taken from along the row axis, two from approximately 30^o^ off the row axis, two from 45^o^ off the row, two from 60^o^ off the row, and two perpendicular to the row.

Soil samples were first passed through a 6 mm sieve to remove stones and root fragments. Nematodes were extracted from 100 cm^3^ subsamples of the freshly sieved soil using a wet-sieving sucrose centrifugation procedure (Forge and Kimpinski, 2007). Plant-parasitic nematodes in each sample extract were counted using a gridded counting dish on an inverted microscope and nematode population density data were expressed on a per 100  cm^3^ soil basis. The population of *Rotylenchus* was identified as *R. robustus* by both morphology and PCR-sequencing of rDNA (accession #MH747474.1, National Center for Biotechnology Information). The population of *Pratylenchus* at the site was previously identified as *P. crenatus* (Zasada et al., 2017). The population of *Paratrichodorus* from a nearby site (500 m) was previously identified as *P. renifer* (Forge et al., 2012).

For samples taken in April, June, and September of 2017, free-living soil nematodes in each sample were counted, approximately 100 were identified to genus, and their population densities were estimated by multiplying relative abundances by the total count. The nematode Enrichment Index (EI), Channel Index (CI), and Structure Index (SI) were computed as originally described by Ferris et al. (2001), and Shannon–Weiner and Simpson indices of diversity (genus level) were computed as described elsewhere (e.g. Wang et al., 2006; Azpilicueta et al., 2014; Pan et al., 2015; Song et al., 2016).

Commencing in 2016, root fragments collected from each sample during the initial sieving were separated into fine (< 2 mm diameter) and coarse (> 2 mm diameter) size fractions, dried and weighed. Fine and coarse root biomass measurements were subsequently expressed on a g root per kg soil basis. The root biomass data were also used to express nematode population densities on a per g root basis in addition to the per 100 cm^3^ basis for analyses of the 2016 to 2019 data.

### Statistical analyses

The nematode population data were subjected to a blocked one-way repeated measures analysis of variance using Proc Mixed in SAS (Statistical Analysis Systems, Cary, NC). Fertilizer N treatment was the fixed factor, block was a random factor in the model, and sample date was considered in the model as repeated measures. Initial analyses were performed with all sample dates in the model. Because *R. robustus* was not recovered from > 50% of plots until after 2015, and because root biomass data collection did not begin until April, 2016, an additional set of analyses were conducted using data from 2016 through 2019. Final analyses of nematode count data were conducted on log-transformed data to minimize heteroscedasticity and improve model fit. Root biomass data were analyzed using the same statistical model used for nematode data. Relationships between nematode population densities and root biomass were explored using correlation and regression analyses. These analyses were performed using the 2016 to 2019 data in entirety and separated by individual sample date. In order to reduce the influence of sample time variation and thereby optimize the potential to assess correlations on the basis of plot-to-plot variation, correlation analyses were also conducted using nematode parameters and root growth measurements averaged over the seven sample dates for each plot.

## Results

### Rotylenchus robustus

**Figure 1:**
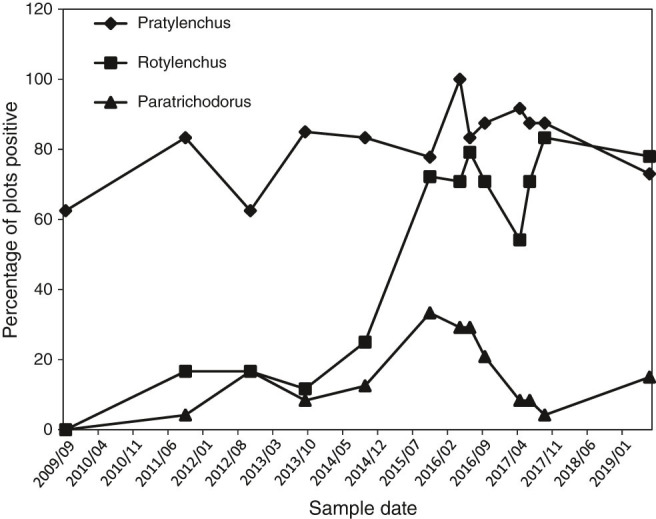
Changes through time in the percentage of samples with *Pratylenchus crenatus*, *Rotylenchus robustus*, and *Paratrichodorus renifer* in the experimental area. At each sample date, a single 100 cm^3^ sample from each of 24 plots (six replicates of four N fertilization treatments) in the experimental area was analyzed. Blueberry bushes were planted in fall of 2008.

**Figure 2:**
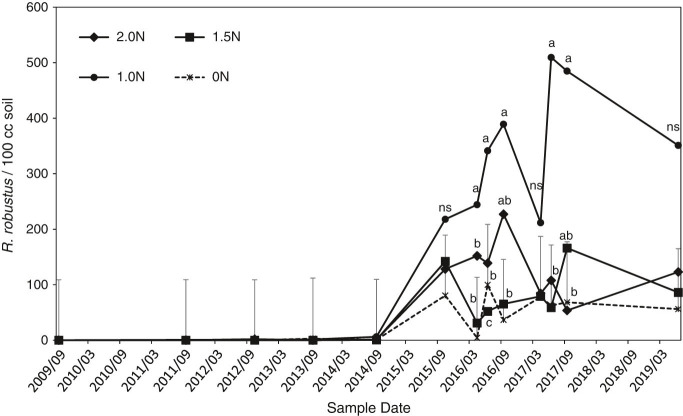
Effects of fertilizer nitrogen application rates on population densities of *Rotylenchus robustus,* from September, 2009 through June, 2019. Error bars are one standard error projecting above the 0N control (pooled standard error from overall ANOVA of non-transformed data). Within a date, points labeled with the same letter are not significantly different (*p* < 0.05) based on Diff procedure in mixed model repeated measures analysis of log-transformed data. NS, no significant differences.

No *Rotylenchus robustus* were observed in the first year of sampling, 2009, but the percentage of plots with detectable populations increased from 11 to 77% between September, 2013 and September, 2015 (Fig. 1). Accordingly, average *R. robustus* population densities also increased dramatically between the September, 2014 and September, 2015 sample dates (Fig. 2). There was a significant N treatment × sample date interaction effect for the 2016 to 2019 data (*p* = 0.02), and the 1N treatment consistently had the greatest *R. robustus* population densities during that period (Fig. 2). Individual-date analyses indicated that *R. robustus* density in the 1N treatment was greater than in the 0N treatment at all dates after 2015 except April, 2017 and June, 2019, and greater than the 1.5N and 2N treatments in June and September of 2017. No other treatments differed from the 0N treatment at any sample date. Main-factor means (averaged over sample dates) were 56, 351, 86, and 123 *R. robustus*/100 cm^3^ for the 0N, 1N, 1.5N, and 2N treatments, respectively. The main-factor mean for the 1N treatment was significantly greater than the 0N treatment (*p* = 0.02) while main-factor means for the 1.5N and 2N treatments did not differ significantly from either the 1N or 0N treatments. For *R. robustus* data expressed on a per g root basis, there was no significant effect of N treatment or N treatment × sample date interaction. The trend with treatment was, however, similar to the trend for *R. robustus*/100 cm^3^ soil data, with main-factor means of 314, 1559, 423, and 334 *R. robustus*/g root in the 0N, 1N, 1.5N, and 2N treatments, respectively.

### Pratylenchus crenatus

**Figure 3:**
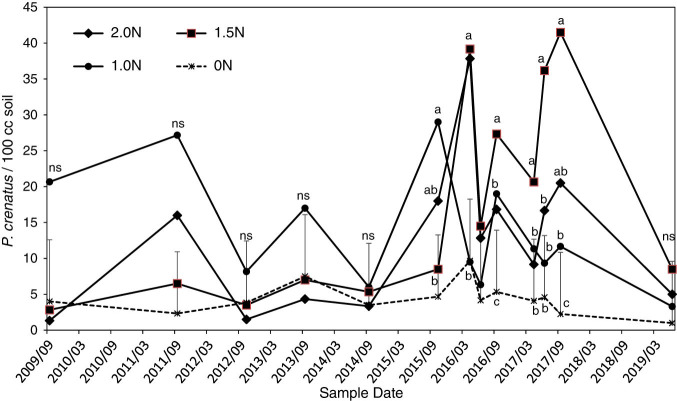
Effects of fertilizer nitrogen application rates on population densities of *Pratylenchus crenatus,* from September, 2009 through June, 2019. Error bars are one standard error projecting above the 0N control (pooled standard error from overall ANOVA of non-transformed data). Within a date, points labeled with the same letter are not significantly different (*p* < 0.05) based on Diff procedure in mixed model repeated measures analysis of log-transformed data. NS, no significant differences.

In contrast to *R. robustus*, the population of *P. crenatus* was present in most plots (62-100%) throughout the study (Figure 1), but population densities seldom exceeded 40 *P. crenatus*/100 cm^3^ soil (Fig. 3). There was a marginally significant main-factor effect of N treatment (*p* = 0.09) and no significant N treatment × sample date interaction on *P. crenatus* population densities when all sample dates were analyzed. For the 2016 to 2019 data there was a significant main-factor effect of treatment (*p* = 0.02) and no N treatment × sample date interaction (Fig. 3). Main-factor means (averaged over 2016-2019 sample dates) were 4, 10, 27, and 17 *P. crenatus*/100 cm^3^ soil for the 0N, 1N, 1.5N, and 2N treatments, respectively. The main-factor mean for the 1.5N treatment was significantly greater than the 0N treatment, with the 1N and 2N treatments not being significantly different from either the 0N or 1.5N treatments. Individual-date analyses indicated that the 1.5N treatment was greater than the 0N control at all sample times after 2015 except June, 2016 and 2019; and the 1N and 2N treatments were greater than the 0N control in September of 2016 and 2017. For *P. crenatus* data expressed on a per g root basis, there was no significant effect of N treatment or N treatment × sample date interaction, but the trend was similar to the trend for *P. crenatus*/100 cm^3^ soil, with main-factor means of 23, 41, 82, and 38 *P. crenatus*/g root in the 0N, 1N, 1.5N, and 2N treatments, respectively.

### Paratrichodorus renifer

*Paratrichodorus renifer* was usually recovered from less than 20% of the plots except for 2015 and 2016 when it was recovered from 20 to 35% of the plots (Fig. 1). Population densities of *P. renifer* remained very low throughout the study, with a maximum of 13 *P. renifer*/100 cm^3^ soil (averaged over all 24 plots sampled) in September, 2016 (data not shown). There was no significant effect of fertilizer N treatment or N treatment × sample date interaction on *P. renifer* population densities, as the low percentage of plots with *P. renifer* resulted in low statistical power to detect treatment effects.

### Root biomass

**Figure 4:**
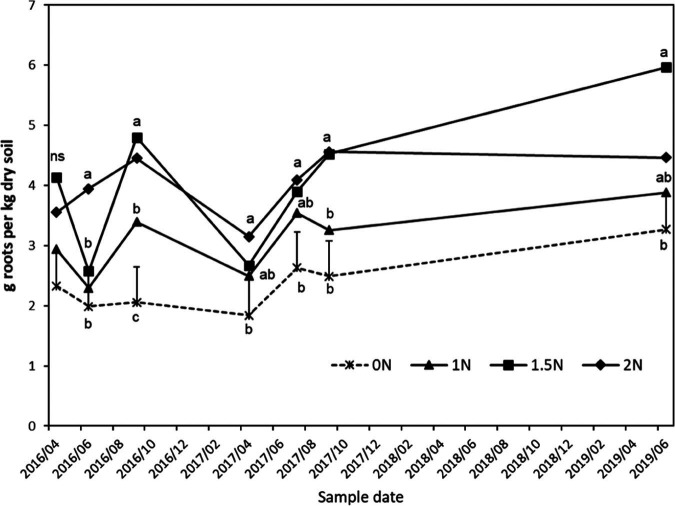
Effects of fertilizer nitrogen rates on the abundance of root fragments extracted from soil cores (g roots per kg dry soil), from April, 2016 through June, 2019. Error bars are one standard error projecting above the 0N control. Within a date, points labeled with the same letter are not significantly different (*p* < 0.05) based on Diff procedure in mixed model repeated measures analysis of log-transformed data. NS, no significant differences.

There was a significant N rate × sampling time interaction for total root biomass (*p* = 0.02; Fig. 4), and the main-factor effect of N rate was significant for both total (*p* ≤ 0.001) and fine (*p* ≤ 0.001; data not shown) root biomass. Total root biomass was generally greater in fertilized treatments than in the 0N treatment (Fig. 4), with overall main-factor means of 2.30, 2.88, 3.68, and 3.60 g roots/kg dry soil for the 0N, 1N, 1.5N, and 2N treatments, respectively. The main-factor mean for the 1N treatment was significantly greater than for the 0N treatment (*p* = 008), and main-factor means for the 1.5N and 2N treatments were significantly greater than the 1N treatment (*p* = 0.03) but did not differ from each other.

**Figure 5:**
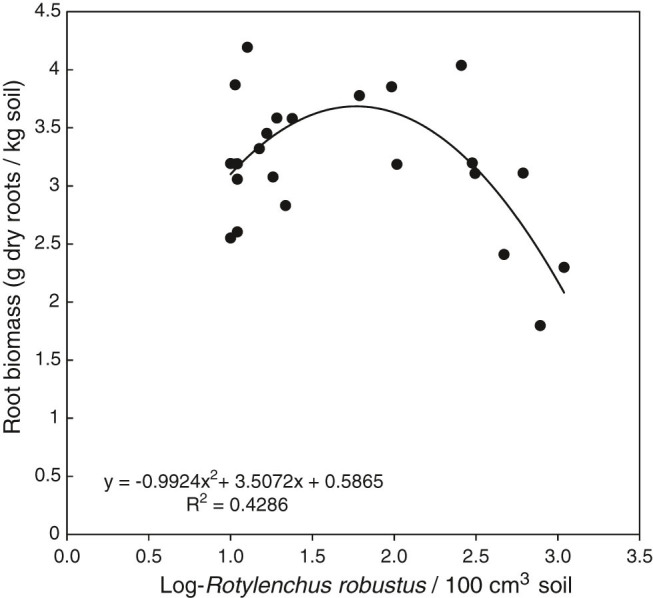
Relationships between *Rotylenchus robustus* population densities and root biomass. Nematode population data and root biomass data for each plot were averages of six sample dates in 2016 and 2017.

Root biomass was not correlated with population densities of any species of plant-parasitic nematode or free-living nematode parameter when correlations were conducted on the entire data set or on individual sample dates. Analyses of relationships across plots, using data averaged over sample dates, revealed a significant quadratic relationship (*p* = 0.003, *r* ^2^ = 0.43) between the logarithm of population densities of *R. robustus* and root biomass (Fig. 5). Plots with very low *R. robustus* population densities had average root biomass values ranging from 2.5 to 4.2 g roots/kg soil while plots with log(*X* + 1) *R. robustus* population densities greater than 2.5 (approximately 300 nematodes/100 cm^3^ soil) had average root biomass values ranging from 1.8 to 3.1 g roots/kg soil (Fig. 5).

### Free-living nematodes

Across sampling dates, the most abundant taxa were *Ditylenchus*, followed by *Acrobeloides, Rhabditidae*, and *Aphelenchoides*, while the most abundant genus in the omnivorous + predacious trophic group was *Clarkus* (Table S1). There was no significant effect of N rate or N rate × sampling time interaction for total free-living nematode abundance, or the abundance of bacterivorous or fungivorous trophic groups (Table 2). For the omnivorous+predacious trophic grouping, the nematode SI, and both indices of genus-level diversity, there was a significant main-factor effect of N rate (*p* < 0.01), but no N rate × sampling time interaction. For all four of these parameters, there was a decrease with N fertilizer rate (Table 2). The N rate × sample time interaction and N rate main-factor effects were both significant (*p* = 0.05 and 0.004, respectively) for the nematode EI. The interaction resulted from the EI being numerically greater in the 0N treatment than fertilized treatments in June and September (Table S1). This trend paralleled the abundance of rhabditid bacterivores which also tended to be more abundant in the 0N treatment than fertilized treatments in June and September (Table S1). Overall means for the EI, reflecting the main-factor effect of N rate, were greater in the 0N treatment than in all fertilized treatments (Table 2). There was no effect of N rate or N rate × sample time interaction on the CI (Table 2).

**Table S1.** Mean (*n*=6 replicate plots) population densities (nematodes/100 cm^3^ soil) of free-living nematodes across dates and N fertilization treatments in 2017.

Date	April 2017				June 2017				September 2017			
Treatment	0N	1N	1.5N	2N	0N	1N	1.5N	2N	0N	1N	1.5N	2N
Ditylenchus	91 (48)	154 (58)	109 (33)	135 (61)	50 (37)	147 (240)	81 (51)	150 (225)	96 (38)	124 (73)	65 (25)	73 (37)
Acrobeloides	43 (43)	104 (77)	69 (31)	98 (80)	41 (28)	166 (286)	117 (54)	109 (97)	31 (10)	77 (51)	81 (24)	68 (68)
Rhabditidae	23 (18)	62 (78)	52 (52)	39 (42)	46 (23)	62 (100)	26 (23)	26 (20)	35 (39)	16 (13)	17 (11)	17 (21)
Aphelenchoides	59 (37)	66 (68)	51 (21)	42 (8)	22 (16)	26 (25)	14 (14)	19 (21)	34 (23)	25 (12)	15 (13)	10 (7)
Filenchus	26 (11)	43 (35)	22 (17)	15 (21)	22 (24)	8 (5)	5 (4)	3 (3)	27 (26)	51 (34)	12 (13)	4 (6)
Cervidellus	23 (23)	51 (47)	28 (39)	14 (17)	13 (25)	42 (72)	5 (8)	4 (7)	16 (13)	23 (16)	8 (4)	5 (6)
Alaimus	9 (10)	7 (7)	3 (6)	1 (2)	21 (18)	7 (7)	13 (10)	5 (6)	24 (16)	15 (19)	5 (5)	4 (7)
Plectus	14 (11)	17 (20)	19 (13)	7 (12)	4 (7)	1 (2)	3 (7)	0	9 (9)	2 (3)	0	2 (5)
Clarkus	4 (10)	7 (12)	2 (3)	0	5 (6)	9 (14)	1 (3)	0	10 (8)	12 (24)	1 (2)	1 (2)
Prismatolaimus	11 (6)	9 (12)	1 (3)	0	3 (5)	0 (1)	0	4 (8)	9 (13))	1 (3)	1 (2)	1 (1)
Aphelenchus	3 (10)	0	1 (3)	1 (2)	1 (1)	6 (15)	0	0	1 (2)	1 (2)	2 (3)	1 (2)
Teratocephalus	6 (9)	1 (2)	1 (3)	3 (5)	0 (1)	1 (2)	0	0	0 (1)	0	0	0
Seinura	1 (1)	0	3 (4)	0	0 (1)	1 (2)	1 (3)	3 (8)	0 (1)	2 (4)	1 (2)	0
Panagrolaimidae	1 (2)	5 (13)	2 (3)	2 (5)	0	0	0	0	0	0	0	1 (2)
Thonus	3 (5)	0	0	0	1 (1)	0	3 (8)	0	1 (3)	2 (4)	0	0
Heterocephalobus	1 (3)	0	0	0	1 (3)	1 (2)	1 (2)	0	1 (2)	0	1 (1)	0
Tylencholaimus	1 (2)	0	0	0	1 (2)	0	0	0	4 (6)	0	0	0
Achromadora	3 (8)	0	1 (3)	0	0 (1)	1 (1)	0	0	0	0	0	0
Cephalobus	0	0	0	0 (1)	1 (3)	0	0	0	1 (3)	2 (4)	0	1 (1)
Monhysteridae	2 (3)	0	0	0	1 (2)	0	0	0	1 (3)	0 (1)	0	0
Diphtherophora	0	0	0	0	0	0	0	0	0	4 (10)	0	0
Diplogasteridae	1 (2)	1 (3)	0	1 (2)	0 (1)	0	0	0	0	0	0	0
Tylenchus	0	0	1 (3)	0	2 (3)	0	0	0	0 (1)	0	0	0
Pungentus	0 (1)	0	0	0	1 (2)	0	2 (5)	0	0	0	0	0
Nygolaimus	0	0	0	0	0	0	0	0	0 (1)	2 (5)	0	0
Acrobeles	0	2 (4)	0	0	1 (2)	0	0	0	0	0	0	0
Rhabdolaimus	0	0	1 (3)	0	0	0	0	0	0 (1)	0	0	0
Aporcelaimellus	0	0	0	0	0	0	0	0	0	1 (2)	0	0
Cylindrolaimus	0 (1)	0	0	0	0	0	0	0	0	0	0	0
Leptonchidae	0 (1)	0	0	0	0	0	0	0	0	0	0	0
Eucephalobus	0 (1)	0	0	0	0	0	0	0	0	0	0	0
Chiloplacus	0 (1)	0	0	0	0	0	0	0	0	0	0	0
Ecumenicus	0	0	0	0	0	0	0	0	0 (1)	0	0	0
Anguinidae	0	0	0	0	0	0	0	0	0 (1)	0	0	0
*Trophic groups (nematodes/100* *cm*^*3*^ *soil) and community structure indices*												
Bacterivorous	138 (98)	259 (214)	179 (45)	165 (123)	133 (78)	282 (452)	165 (56)	148 (94)	128 (72)	137 (69)	112 (29)	98 (47)
Fungivorous	180 (60)	262 (106)	185 (43)	193 (68)	96 (61)	186 (276)	99 (48)	171 (242)	162 (66)	204 (96)	94 (37)	89 (44)
Omni+Preds^a^	8 (11)	7 (12)	5 (4)	0	6 (6)	10 (15)	8 (9)	3 (8)	12 (8)	17 (28)	2 (2)	1 (2)
Enrichment Index	51 (9)	51 (10)	55 (12)	53 (10)	67 (12)	52 (11)	46 (15)	53 (8)	56 (10)	46 (4)	45 (12)	46 (11)
Structure Index	41 (10)	25 (15)	23 (12)	12 (11)	54 (19)	32 (14)	29 (11)	17 (13)	52 (13)	46 (10)	21 (14)	13 (13)
Channel Index	66 (19)	61 (27)	50 (21)	60 (24)	30 (17)	50 (21)	54 (21)	51 (31)	56 (19)	70 (18)	58 (12)	64 (25)
Shannon Diversity	7.30 (1.11)	5.66 (1.65)	5.97 (0.88)	4.66 (1.15)	6.79 (1.15)	6.46 (1.22)	4.94 (1.25)	3.99 (0.80)	7.51 (1.92)	7.33 (2.48)	5.37 (1.09)	4.67 (1.01)
Simpson Diversity	5.51 (1.04)	4.60 (1.58)	4.72 (0.70)	3.82 (0.98)	5.42 (1.00)	5.12 (1.10)	3.91 (1.07)	3.17 (0.73)	5.78 (1.79)	4.69 (1.80)	4.24 (1.02)	3.69 (0.84)

**Notes:** Values in parentheses are standard deviations (*n* = 6). Taxa are arranged in order of decreasing overall abundance. ^a^Omni + Preds = Omnivorous + predacious nematodes combined.

**Table 2.** Effects of nitrogen fertilizer treatments on the Shannon–Weiner (S–W) and Simpson (Simpson) indices of genus-level diversity, the nematode Structure Index (SI) and Enrichment Index (EI), and population densities (nematodes/100 cm^3^) of omnivorous+predacious (O + P), bacterivorous (Bact) and fungivorous (Fung) trophic groups.

Treatment	S–W	Simpson	SI	EI	CI	O + P	Bact	Fung
0N	7.02a^a^	5.57a	49a	58a	51	9ab	133	146
1N	6.48a	4.90ab	35b	49b	60	11a	226	218
1.5N	5.43b	4.36b	24c	49b	54	5bc	152	126
2N	4.44c	3.51c	14d	50b	58	1c	137	151
*ANOVA summary (p-values)*^*b*^								
Treatment	< 0.001	< 0.001	< 0.001	0.004	0.39	0.004	0.18	0.09
Date	0.21	0.57	0.05	0.07	0.002	0.47	0.19	0.04
T × D	0.57	0.80	0.29	0.05	0.18	0.67	0.91	0.70

**Notes:** Data are means of three sample dates (April, June, September) and six replicate plots per treatment. ^a^Means within a column labeled with the same letter are not significantly different according to Duncan’s means separation calculated at *p* = 0.05. ^b^Mixed-model repeated measures analysis of variance.

## Discussion

The field experiment was established to assess blueberry yield responses to a range of N fertilization rates of interest to growers in the region. While the N fertilization rates were not fixed, the experimental design resulted in three distinct levels of cumulative N fertilizer inputs over 11 years that span the range of N fertilization rates used by organic (0N) and conventional (1N, 1.5N, and 2N) blueberry growers in the region.

In early years of nematode sampling (2009 through 2014), populations of *Rotylenchus robustus, Pratylenchus crenatus*, and *Paratrichodorus renifer* were all detected in the experimental plots, but the frequencies of occurrence among plots and population densities of both *R. robustus* and *P. renifer* were very low. By the end of 2015, *R. robustus* was detected in over 70% of plots with overall mean population densities (averaged over all plots) in excess of 150 *R. robustus*/100 cm^3^ soil. It is relevant to note that this nematode became a contaminant in an earlier and nearby field micro-plot experiment, where it quickly reached comparably high population densities feeding on blueberry cultivar Chippewa (Forge et al., 2012), further illustrating the host status of blueberry for *R. robustus*.

The lack of *P. renifer* population buildup in these plots, regardless of N fertilization rates, was unexpected as we had previously determined that highbush blueberry is a good host for this species in a nearby field micro-plot study with the same soil type (Forge et al., 2012). A major difference between the micro-plot study and this field experiment is that soil in the micro-plots was fumigated prior to planting, suggesting that biological antagonists in the non-fumigated soil at the field site could have influenced population dynamics of *P. renifer*.

Although blueberry is relatively shallow rooted for a woody perennial (Trehane, 2004), it is unclear if our sampling, which was limited to 30 cm depth, allowed us to adequately represent the plant-parasitic nematode populations in this study. Relatively little is known of the preference for particular soil horizons or depths of *R. robustus, P. crenatus*, or *P. renifer* under perennial crops. Boag (1982) reported that *R. robustus* was most abundant at 10 to 20 cm in a spruce nursery, and Forge et al. (1998) reported that *P. penetrans* was most abundant at 15 cm under red raspberry. These two studies suggest that sampling to 30 cm was likely adequate to represent the populations of *R. robustus* and *P. crenatus*, respectively, assuming that *P. crenatus* has similar ecological preferences as *P. penetrans*. In contrast, the vertical distribution of *P. minor* in annual cropping systems is known to vary seasonally and at times to be concentrated at depths well below 30 cm (McSorley and Dickson, 1990; Perez et al., 2000). Assuming similar ecological preferences for *P. renifer*, such results suggest that our sampling strategy could have missed a significant portion of the *P. renifer* population. Future research to document seasonal variation in the vertical distribution of these nematode species in relation to blueberry roots would improve understanding of their population dynamics and potential impacts on blueberry.

An additional limitation of our analysis of *P. crenatus* population dynamics is that we did not assess population densities of the nematode in blueberry roots. Previous research, including samples from this site, demonstrated that *P. crenatus* does colonize blueberry roots, but that population densities in roots were generally quite low relative to most other lesion nematode-host relationships (Zasada et al., 2017). When sampling over multiple years, substantial changes in *Pratylenchus* soil population densities, due either to time or a treatment, generally reflect changes in the size of the overall population, roots inclusive (e.g. Vrain et al., 1997; Forge and Kempler, 2009). We suggest therefore that while the lack of data on *P. crenatus* in root tissue limits our ability to make inferences about levels of parasitism per se, our data on *P. crenatus* population densities in soil are nonetheless indicative of the influences of time and N fertilization treatments on the overall population density of *P. crenatus*.

### Effects of N fertilization

The positive responses of *P. crenatus* and *R. robustus* populations to N fertilization are consistent with earlier studies describing the effects of N fertilization on populations of plant-parasitic nematodes in grassland, forage, and annual production systems (Dmowska and Ilieva, 1995; Todd, 1996; Sarathchandra et al., 2001; Verschoor et al., 2001; Forge et al., 2005a; Wang et al., 2006), and a more recent study of the effects of N fertilization rates on *Mesocriconema xenoplax* on grapevine (Forge et al., 2019). However, in this study, the increase in population densities with N fertilization rate was not unimodal. Population densities attained in the 2N treatment tended to be lower than those attained in the 1N and 1.5N treatments for *R. robustus* and *P. crenatus*, respectively. Reasons for the lack of continuous increase in population densities with N fertilization rate are unclear. We speculate that direct ammonia/ammonium toxicity or salt stress resulting from the fertilizer applications could have suppressed additional population growth of these species in the 2N treatment. Indeed, relatively high (> 200 kg N/ha) single-dose applications of ammoniacal fertilizers have been used to suppress populations of plant-parasitic nematodes prior to planting annual crops (Rodriguez-Kabana, 1986; Oka et al., 2006; Su et al., 2015). Electrical conductivity of the soil solution increased with N fertilization rate, with values near 800 μS cm^–1^ for the broadcast-applied 2N treatment in 2015 (Messiga et al., 2018). As the EC measurements were taken in fall of each year, approximately 4 months after fertilizer was applied, it was not possible to determine maximum salt levels to which nematodes would actually have been exposed. However, for the treatments evaluated in this study, the annual fertilizer application was split into three separate applications over an eight-week period, and the fertilizer was broadcast onto the sawdust mulch. These application practices would have dampened movement of mineral N into the soil, minimizing the likelihood that toxic levels of ammonium, nitrate, or total salts would accumulate in soil solution. Another possibility is that soil pH became a stress for nematodes in the 1.5 N and 2 N treatments. Soil pH was not measured on samples taken for nematode analyses, but our previous analyses indicated that soil pH was reduced from 5.13 in the 0N treatment to 4.69 in the 2N broadcast treatment by 2015 (Messiga et al., 2018). It is worth noting that berry yields were suppressed in this field experiment in the 2N treatment relative to the 1N and 1.5N treatments, particularly when the fertilizer was applied through the irrigation line (fertigated), and this effect was attributed to high salt levels and low soil pH (Messiga et al., 2018).

### Relationships with root biomass and plant vigor

We suggest that the positive influences of N fertilization on *R. robustus* and *P. crenatus* were mediated through enhanced availability of roots, as root biomass increased with N fertilization roughly in parallel with the increased nematode population densities. A related possibility is that improved N status of root tissues, independent of changes in root biomass per se, resulted in improved nematode fecundity. The fecundity of herbivorous arthropods is responsive to the N status of host plant tissues (Mattson, 1980), and we hypothesize that this also applies to plant-parasitic nematodes. We did not directly measure the N status of root tissue so it is not possible to disentangle the relative effects of increased root availability and changes in nutritional value of the root tissue. In other crops such as grape, root tissue nitrogen concentrations are known to increase with fertilization (Pino et al., 2012).

The substantial plot-to-plot variation in *R. robustus* population densities provided an opportunity to assess relationships between *R. robustus* population densities and root biomass at the site. These plot-to-plot analyses revealed a curvilinear relationship between time-averaged *R. robustus* population densities and root biomass, with smallest root biomass values observed in plots with the largest *R. robustus* population densities. It is unclear if this apparent effect of *R. robustus* on root biomass will translate to an effect on productivity. Berry yields through 2015 for this experiment have been reported elsewhere (Ehret et al., 2014; Messiga et al., 2018). In parallel with *R. robustus* and *P. crenatus* population densities, berry yields increased with fertilization but without clear differences between 1N, 1.5N, and 2N broadcast treatments (Ehret et al., 2014; Messiga et al., 2018). Thus, at the level of comparison across treatments at this site, there is no clear relationship between *R. robustus* population densities and berry yield. For perennial fruit crops, fruit production is affected by numerous factors besides overall plant vigor, including plant nutrient status the preceding fall, winter chilling unit accumulation, pruning and crop load management, and pollination in spring (Trehane, 2004). Year-to-year variation in such factors can obscure relationships between plant-parasitic nematode population densities and yields of perennial fruit crops. Controlled inoculation greenhouse and field micro-plot studies such as those used by Forge et al. (2012) are needed to confirm the damage potential of *R. robustus* on highbush blueberry.

### Free-living soil nematodes

Reductions in the abundance of omnivorous + predacious nematodes, the nematode Structure Index (SI), and both indices of diversity (S-W and Simpson) with N fertilizer rate are consistent with results from previous studies of N fertilization of forage and annual cropping systems (e.g. Forge et al., 2005b; Wang et al., 2006; Azpilicueta et al., 2014; Pan et al., 2015; Song et al., 2016). They are also consistent with experimental work documenting the greater sensitivity of omnivorous and predacious nematodes in the family Dorylaimida to dissolved nitrate and ammonium (Tenuta and Ferris, 2004).

The overall abundance of omnivores and predators in the Dorylaimida in this blueberry planting was remarkably low compared to other studies. For example, an earlier study of nematode communities in a nearby (1 km) field of forage grasses on the same soil type reported omnivorous and predacious nematode population densities in the range of 50 to 150 nematodes/100 cm^3^ soil (Forge et al., 2005b). The overall abundance of bacterivorous and fungivorous nematodes in this blueberry experiment did not differ as markedly from other studies. The relatively low abundance of omnivorous and predacious nematodes suggests that some aspect of the blueberry production system may be inhibitory to omnivorous and predacious nematodes. Possibilities include low soil pH, sawdust mulch, or perhaps exudates from the roots and ericoid mycorrhizae. Given the importance of omnivorous and predacious nematodes to soil food webs, additional research to further document the suppression of these nematodes under blueberry production could contribute to improved understanding of the impacts of blueberry production on overall soil health.

The response of the Enrichment Index (EI) ran counter to hypothesized, with all fertilized treatments having lower EI values than the 0N treatment, and with no statistically significant differences between the 1N, 1.5N, or 2N treatments. This trend in the EI was primarily a reflection of rhabditid bacterivores, the only cp-1 enrichment opportunists at the site, being more abundant in the 0N treatment at two of the three sample dates.

High values for the nematode SI, various indices of diversity and abundances of omnivorous, and predacious nematodes are indicative of highly structured soil food webs that have been proposed to have enhanced potential for regulation of parasitic nematode populations (Sánchez-Moreno and Ferris, 2007; McSorley et al., 2008; Ferris et al., 2012). The decreases in these parameters with N fertilization rate are consistent with the hypothesis that N inputs may facilitate buildup of plant-parasitic nematode populations by reducing soil food web complexity and the abundance of nematode antagonists. Additional research using laboratory assays of general biological suppression (Timper, 2014) with soil samples from fertilized and non-fertilized plots would help determine if reduced food web complexity and prevalence of nematode antagonists contributes to the development of larger plant-parasitic nematode population densities in fertilized soils.

### Conclusions

Populations of *R. robustus* and *P. crenatus* both responded positively, relative to unfertilized controls, to N fertilization at rates within the range of rates typically used by blueberry growers in the region. Population densities did not increase monotonically with N fertilizer rate for either species, however, suggesting that greater N fertilization rates could be suppressive to plant-parasitic nematodes. Because berry yields also increased between the non-fertilized control and 1N treatment, but with minor differences between the 1N, 1.5N, and 2N broadcast treatments (Ehret et al., 2014; Messiga et al., 2018), our data do not point to an optimal rate of N fertilization that would provide high yields while minimizing buildup of *R. robustus* or *P. crenatus*.

Indices of free-living nematode community structure, specifically the nematode Structure Index and two indices of diversity, declined monotonically with N fertilizer rate. These results indicate broader changes in the soil food web that have been associated with reduced activity of antagonists of plant-parasitic nematodes and could in-turn have contributed to the increased population densities of *R. robustus* and *P. crenatus* in fertilized plots relative to non-fertilized plots. Additional research incorporating bioassays of soil suppressiveness would help determine the importance of such interactions, relative to improved abundance and nutritional quality of host roots, as factors contributing to N fertilization-induced increases in population densities of plant-parasitic nematodes.
